# Establishment and evaluation of an *in vitro* blast lung injury model using alveolar epithelial cells

**DOI:** 10.3389/fpubh.2022.994670

**Published:** 2022-12-23

**Authors:** Chunjie Ding, Shan Hong, Miao Zhang, Yunzhe Sun, Ning Li, Jing Zhang, Lan Ma, Linqiang Tian, Wenjie Ren, Lin Zhang, Sanqiao Yao

**Affiliations:** ^1^School of Public Health, Xinxiang Medical University, Xinxiang, China; ^2^School of Public Health, North China University of Science and Technology, Tangshan, China; ^3^School of Public Health, Weifang Medical University, Weifang, China; ^4^Institute of Trauma and Orthopedics, Xinxiang Medical University, Xinxiang, China; ^5^Clinical Medical Research Center for Women and Children Diseases, Maternal and Child Health Care Hospital of Shandong Province Affiliated to Qingdao University, Jinan, China

**Keywords:** gas explosion, blast injury, alveolar epithelial cells, lung injury model, oxidative stress response

## Abstract

**Background:**

Gas explosion is a fatal disaster commonly occurred in coal mining and often causes systematic physical injuries, of which blast lung injury is the primary one and has not yet been fully investigated due to the absence of disease models. To facilitate studies of this field, we constructed an in vitro blast lung injury model using alveolar epithelial cells.

**Methods:**

We randomly divided the alveolar epithelial cells into the control group and blast wave group, cells in the blast wave group were stimulated with different strengths of blast wave, and cells in the control group received sham intervention. Based on the standards we set up for a successful blast injury model, the optimal modeling conditions were studied on different frequencies of blast wave, modeling volume, cell incubation duration, and cell density. The changes of cell viability, apoptosis, intracellular oxidative stress, and inflammation were measured.

**Results:**

We found that cell viability decreased by approximately 50% at 6 h after exposing to 8 bar energy of blast wave, then increased with the extension of culture time and reached to (74.33 ± 9.44) % at 12 h. By applying 1000 ~ 2500 times of shock wave to 1 ~ 5 × 105 cells /ml, the changes of cell viability could well meet the modeling criteria. In parallel, the content of reactive oxide species (ROS), malonaldehyde (MDA), interleukin 18 (IL-18), tumor necrosis factor alpha (TNF-α), and transforming growth factor beta (TGF-β) increased in the blast wave group, while superoxide dismutase (SOD) and Glutathione -S- transferase (GST) decreased, which were highly consistent with that of human beings with gas explosion-induced pulmonary injury.

**Conclusion:**

An in vitro blast lung injury model is set up using a blast wave physiotherapy under 8 bar, 10 Hz blast wave on (1 ~ 5) ×105 alveolar epithelial cells for 1 000 times. This model is flexible, safe, and stable, and can be used for studies of lung injury caused by gas explosion and blast-associated other external forces.

## 1. Introduction

Coal is an important source of energy on earth, and the modernization of coal mining varies between countries (1, 2). China is the one who has abundant coal reserves and has made great breakthroughs over the past years in coal mining and utilization (3). However, coal mine accidents do occur frequently, mainly including gas explosion accidents, mine water accidents and poisoning suffocation accidents, of which gas explosion accidents contribute to a large proportion (4). Generally, the occurrence of gas explosion is mainly due to poor ventilation in coal mining, where gas concentration can quickly climb to 5–15% and, after mixing with about 12% oxygen in the air, self-accelerating reaction will occur under the induction of open fire, leading to explosion (5). Recent data released by the National and Provincial Coal Mine Safety Administration shows that there are 272 coal mine gas fatalities from 2010 to 2019 with 1952 deaths, including 107 gas outburst accidents with 735 deaths and 165 gas explosion, combustion and suffocation accidents with 1,217 deaths. It is apparent that coal mine accidents mainly gas explosions have brought severe threats to lives of coal miners and national economy, and it is urgent to find out solutions for the loss.

The main damage factors of gas explosion injuries include blast wave, flame temperature, and poisonous gas, of which blast injuries contribute to a large proportion (6, 7). The blast wave could impact severely on fluid containing organs, including the lung (8). Among all damages caused by blast wave, lung damage is one of the most common causes of death (9). When the lung is exposed by blast wave, a series of physiological stress responses such as oxidative stress, inflammation, and edema often occur (10–12). Moreover, some researchers used mice to establish a blast lung injury model *in vivo* based on the shock tube to explore the mechanism of blast lung injury, and found that the blast wave could cause inflammation, oxidative stress and apoptosis in the lung tissues of mice (13, 14). Mechanistically, damage in alveolar epithelial cells is the main cause of oxidative stress and inflammatory response, it plays an important role in blast wave-induced lung injury (15). Therefore, a lung injury model based on alveolar epithelial cell is particularly useful for mechanistic studies, especially considering the high cost and difficulties in energy control in establishing animal blast lung injury models (16). Herein, to establish a stable, reliable and flexible *in vitro* model, we exposed the alveolar epithelial cells with specified energies of blast wave and examined the appearance of blast injury.

The *in vitro* model prepared by our method can be used for studying the pathogenesis of blast wave-caused injury, especially those occurred in lung tissues. Also, it highlights emerging directions for studies concerning blast injury in different parts of the body, and the injuries may not be caused by blast wave, but similar external forces, such as brain blast injury, liver and spleen blast injury (17–19). Moreover, this model can also be used to observe the development of blast lung injury *in vitro*, so as to systematically study the molecular mechanism of related physical damages, which could contribute to revealing the pathogenesis and clinical treatment of blast lung injury.

## 2. Materials and methods

### 2.1. Materials

The alveolar epithelial cell line L2 cells and A549 cells were purchased from Otwo Biotech (Shenzhen, China). Fetal bovine serum (FBS) was provided by ExCell Bio (Jiangsu, China). High glucose Dulbecco's modified Eagle's medium (H-DMEM), MDA kit, SOD kit, Annexin V-FITC/PI kit, GST kit and antibodies including IL-18, TNF-α, TGF-β were obtained from Solarbio (Beijing, China). Cell counting kit 8 (CCK-8) was bought from DOJINDO (Beijing, China). Calcein/PI cell viability assay kit and ROS assay kit were purchased from Beyotime (Shanghai, China).

### 2.2. Cell culture and model building

**Figure 1:**
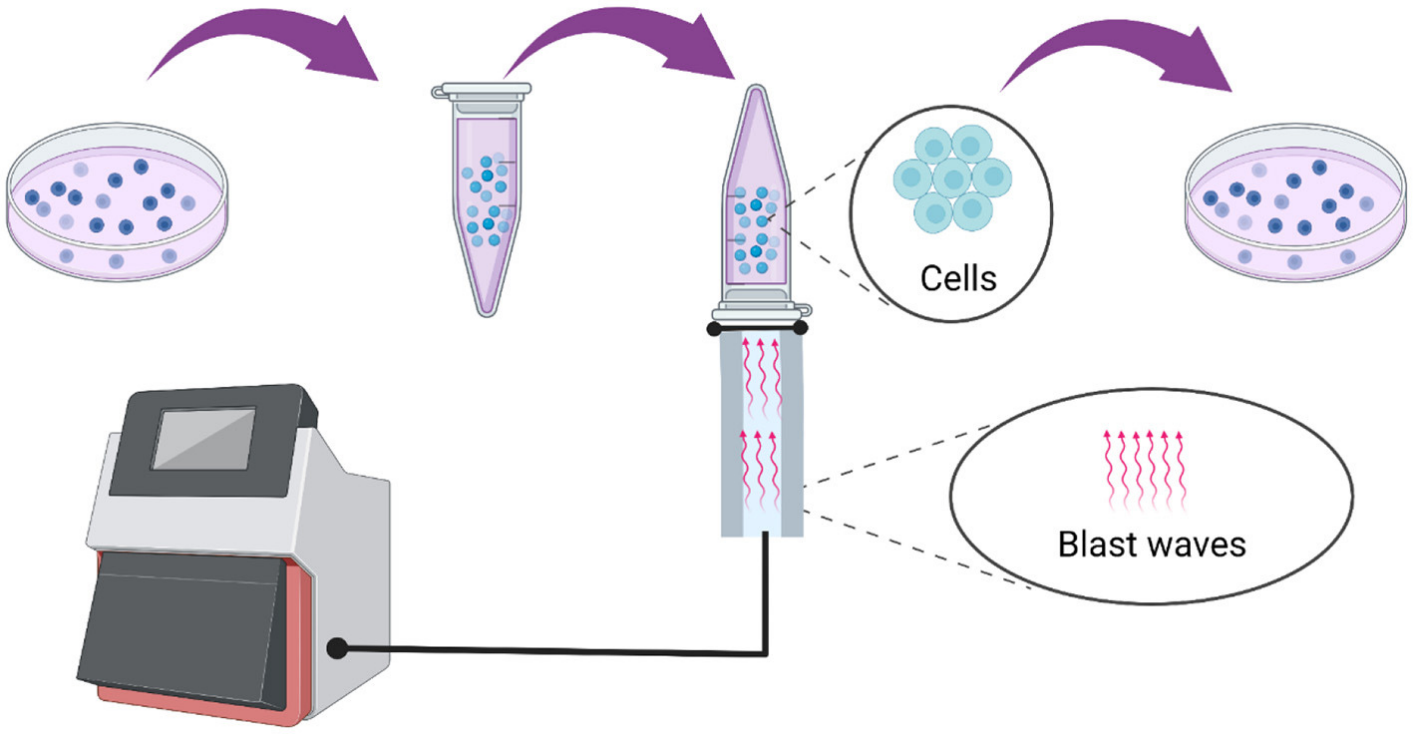
Model construction process using a blast wave therapy instrument type HM08CJ based on the alveolar epithelial cells.

As shown in Figure 1, the alveolar epithelial cells were cultured in H-DMEM with 10% FBS containing 100 U/mL penicillin-streptomycin under the standard culture conditions (37°C and 5% CO_2_). The medium was discarded when the cell confluence reached 70–80%, followed by washing with 1 × PBS twice. Then, the cells were digested with trypsin-EDTA for 2 min, and the digestion was terminated by adding adequate volume of complete medium, followed by beaten gently until they completely fell off. Further, the cells were counted and a cell suspension with a volume of 1 mL and a concentration of 1 × 10^5^/mL was prepared and placed in a 5 mL centrifuge tube. Then, the tube was sealed with aseptic sealing film and inverted on the top of the warhead on a blast wave therapy instrument, and the coupling agent was applied to the middle joint. Subsequently, the blast wave physiotherapy was used for modeling and matched with normal cells. After stimulation, the cells were seeded in a 96-well plate at different concentrations per the requirements of follow-up experiments. Besides, the 5 mL centrifuge tube was used for each model construction and the fixed frequency of the blast wave was 10 Hz.

### 2.3. Experimental groups

Six factors that may influence the model establishment were considered and studied. To determine the optimal blast wave energy on cell viability, we set six energy gradients of 0.5, 2, 4, 6, 8, and 10 bars, and the control group was matched. In parallel, six time points of 4, 6, 8, 10, 12, and 24 h were set to find the optimal culture time, five impulse frequencies of 500, 1,000, 1,500, 2,000, and 2,500 were utilized to find the appropriate impulse frequency, five modeling volumes of 1, 2, 3, 4, and 5 mL were set in a 5 mL centrifuge tube to determine the most appropriate modeling volume, six modeling concentrations of 1, 2, 4, 5, 7.5, and 10 × 10^5^/mL were set to search for the ideal modeling concentration, and five cell plating concentrations of 2 × 10^3^, 4 × 10^3^, 6 × 10^3^, 8 × 10^3^, and 1 × 10^4^ cells/well were set to identify the optimal cell plating number. Each group was repeated with 5 samples in parallel, and each experiment was repeated at least 3 times.

### 2.4. Cell viability assay

The cells were randomly divided into two groups: the control group and the blast wave group, each with 5 samples in parallel, and each experiment was repeated at least 3 times. Cells in the blast wave group were exposed to 0.5, 2, 4, 6, 8, and 10 bars of blast wave. On the completion of cell stimulation, 10 μL of CCK-8 solution was added to each well, followed by incubation for 2 h at 37°C (20). The absorbance was measured at 450 nm by a multi-mode plate reader (Bio Tek, VT, USA).

### 2.5. Settlement of a standard for *in vitro* blast lung injury model

The standards for establishment of the *in vitro* blast lung injury model were set according to the requirements of toxicology. First, the viability of adherent cell should decrease to (50 ± 5)% at 6 h after blast wave stimulation. Secondly, the viability of cells should increase to no more than 75% after 12 h of maintenance.

### 2.6. Calcein-AM/PI assay

After stimulation, the cells were seeded in a 96-well plate and the medium was discarded after 6 h of maintenance, followed by washing with 1 × PBS twice. Then, 100 μL/well of Calcein/PI detection solution was added, followed by incubation for 30 min at 37°C in darkness. The cells were imaged using the Image X press ^®^ Micro Confocal system (Molecular Devices, CA, USA), where the living cells were stained in green with Calcein, and the membrane-ruptured cells were stained in red with PI. Each group was repeated with 10 samples in parallel, and each experiment was repeated at least 3 times.

### 2.7. ROS detection assay

The cells were cultured and stimulated in line with above steps. According to the manufactures' instructions, the intracellular ROS was detected after 6 h of maintenance using a fluorescence probe hybridization with DCFH assay kit, and the fluorescence intensity of ROS was observed under fluorescence microscope upon the completion of above stimulations. Similarly, the contents of MDA, SOD, and GST in cells were also detected after 6 h of maintenance using specific kits per the manufacturer's instructions. Each group was repeated with 10 samples in parallel, and each experiment was repeated at least 3 times.

### 2.8. Flow cytometry assay

The cells were stimulated in line with above steps and cultured for 6 h, followed by washing with 1 × PBS twice. Then 1 × 10^6^ cells were separated and resuspended in 400 μL of Annexin V-FITC binding solution. 5 μL of Annexin V-FITC and 5 μL of PI were added to the cells in order and the stained cells were subjected to the Accuri C6 flow cytometer (BD Biosciences, CA, USA). Data acquisition and analysis were performed using the BD software. Each group was repeated with 10 samples in parallel, and each experiment was repeated at least 3 times.

### 2.9. Immunofluorescence

The cells were stimulated in line with above steps and seeded on a round pick in a 24-well plate (3 × 10^5^/well). After maintained at 37°C for 6 h, the cells were fixed with 4% formaldehyde for 10 min and penetrated with 0.3% Triton X-100 for 10 min at room temperature. Then, the cells were blocked with goat serum for 30 min and coated with primary antibodies of IL-18, TNF-α and TGF-β. After incubation for about 12 h at 4°C, the cells were incubated with secondary antibody conjugated by TRITC or FITC for 50 min. Then the cells were incubated with DAPI solution for 10 min. Finally, the cells were imaged under the Image Xpress ^®^ Micro Confocal system. Each group was repeated with 10 samples in parallel, and each experiment was repeated at least 3 times.

### 2.10. Statistical analysis

Each experiment was repeated at least 3 times. All experimental data were analyzed and visualized using GraphPad Prism 7.0 and expressed as mean ± standard deviation. One-way *ANOVA* was applied for multi-group comparison followed by an *SNK post-hoc* test, and *t-*test was used for comparison between two independent groups. A *P*-value < 0.05 was considered statistically significant.

## 3. Results

### 3.1. L2 cell viability decreases in a blast energy and post-blast culture time dependent manner

**Figure 2:**
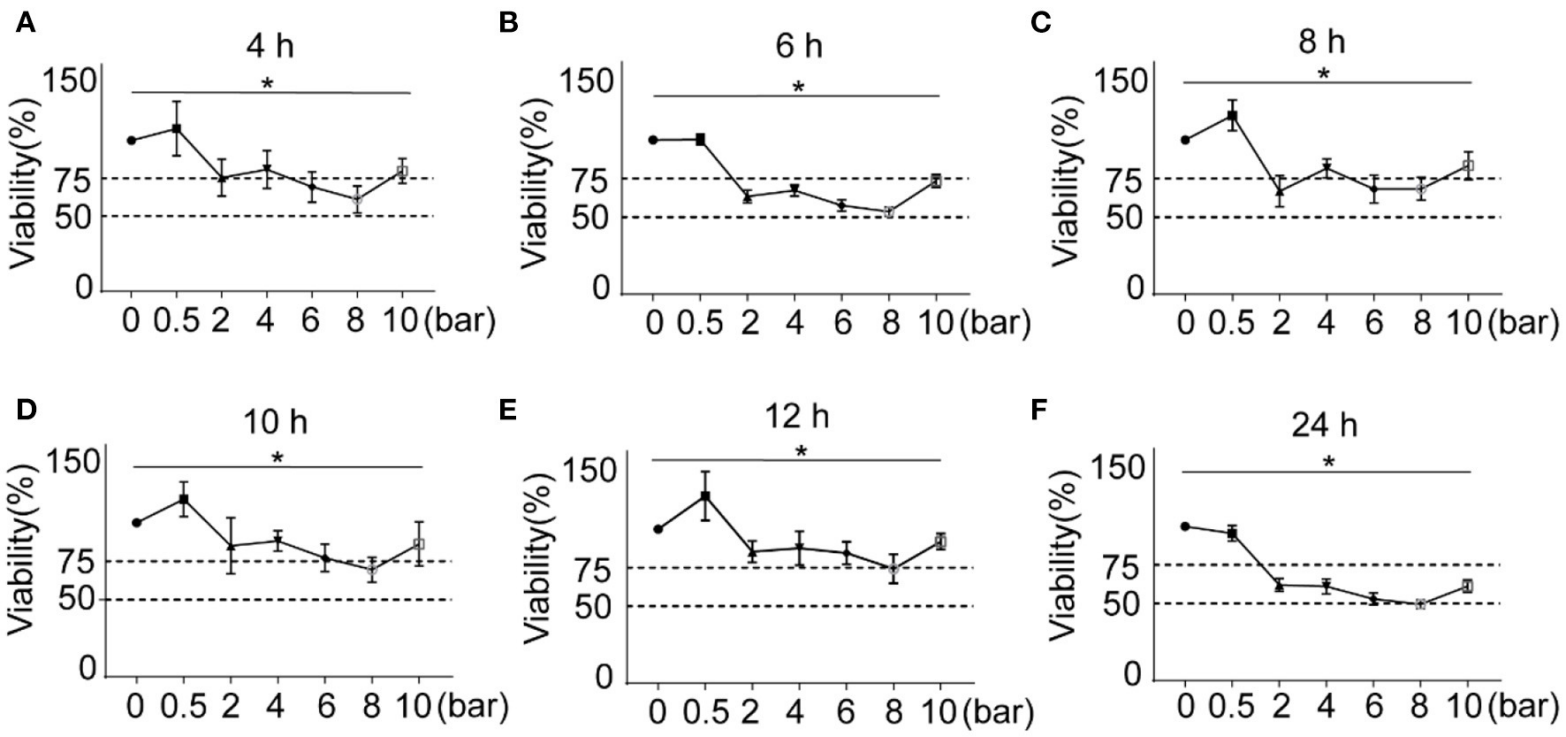
Effects of different blast wave energy and post exposure culture time on L2 cell viability. **(A–F)** show the changes of cell viability after 4, 6, 8, 10, 12, and 24 h post exposure culture time. Impulse energy gradient is set at each time point for comparison. Data represent mean ± standard deviation; *n* = 5. *P < 0.05.

As shown in Figures 2A–F, the cells were stimulated with 0.5, 2, 4, 6, 8, and 10 bars of blast wave, after incubation for 4, 6, 8, 10, 12, and 24 h, the cell viability increased at 0.5 bar energy in varying degrees except for 24 h group. In comparison, the cell viability of 24 h time point decreased in an energy dependent manner. According to criteria set for blast modeling, an 8-bar energy of blast wave introduced a reduction of cell viability to (53.52 ± 2.86)% at 6 h and (74.33 ± 9.44)% at 12 h, other energies of the same incubation period caused lower reduction of cell viability as compared to the 8-bar group. Therefore, 8-bar blast wave energy and 6 h culture time were determined for the preparation of blast lung injury cell model.

### 3.2. L2 cell viability decreases with increased blast frequency

**Figure 3:**
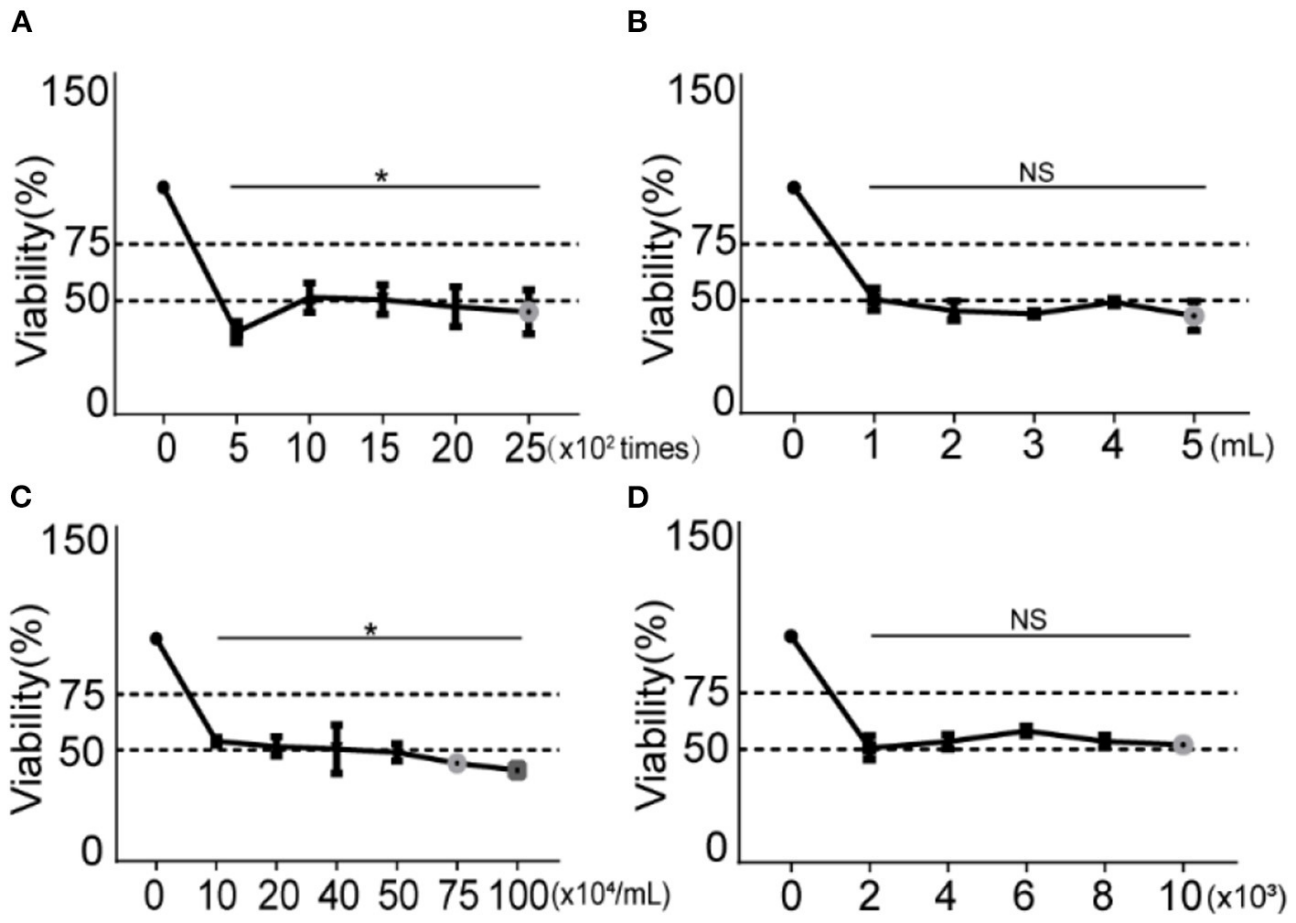
Changes of cell viability under different blast wave frequencies, modeling volumes, modeling concentrations and cell plating number. **(A)** Different impulse frequencies are set for comparison. **(B)** Different modeling volumes are set for comparison. **(C)** Different modeling concentrations are set for comparison. **(D)** Different cell plating number is set for comparison. Group 0 represents the control group and data represent mean ± SD; *n* = 5. *P < 0.05. NS, not significant, *P* > 0.05.

As shown in Figure 3A, the cells were stimulated by 500, 1,000, 1,500, 2,000, and 2,500 impulses at a fixed frequency of 10 Hz, 8-bar blast energy, and 6 h post exposure culture time. Compared with the control groups, the cell viability reduced with increased blast frequency, the trough of (36.28 ± 4.61)% appeared in 500-impulse group, and half number of cell death was found in 1,000, 1,500, 2,000, and 25,000-impulse groups. According to the established model making standard, the impulse between 1,000 and 2,500 is available for the preparation of blast lung injury cell model.

### 3.3. The modeling volume has no effect on cell viability

To examine the effect of modeling volume on cell viability under constant cell concentration, five modeling volumes (1, 2, 3, 4, and 5 mL) were set. As shown in Figure 3B, comparing with the control group, the cell viability of all modeling volumes decreased, but the differences between them were of no statistical significance.

### 3.4. Increased cell modeling concentration is associated with decreased cell viability

As shown in Figure 3C, the cell modeling concentration was adjusted to 1, 2, 4, 5, 7.5, and 10 × 10^5^/mL. After being stimulated with 8-bar blast wave and 1,000-impulse at 10 Hz in 1 mL complete medium, 1 × 10^4^ cells/well were plated in a 96-well plate and incubated for 6 h. Compared with the control group, the cell viability decreased at all modeling concentrations and showed a concentration-dependent trend. Referring to modeling criteria, a reduction of ~50% of cell viability is determined in concentration groups of 10, 20, 40, and 50 × 10^4^ cells [(53.95 ± 1.71), (51.58 ± 4.31), (50.41 ± 10.96), and (48.97 ± 3.86)%], which are all available in establishing blast lung injury models.

### 3.5. The cell plating number has no effect on cell viability

To explore the effect of different cell plating number, we set five gradients of 2, 4, 6, 8, and 10 × 10^3^/well in a 96-well plate. The cells were stimulated with fixed conditions of 8-bar blast wave, 1,000-impulse at 10 Hz in 1 mL complete medium, 1 × 10^5^/mL modeling concentration, and 6-h post stimulation culture time. As shown in Figure 3D, the cell viability decreased in all plating number groups as compared to the control, but the differences between different stimulation groups were of no statistical significance.

### 3.6. Effects of specific modeling conditions on alveolar epithelial cells

**Figure 4:**
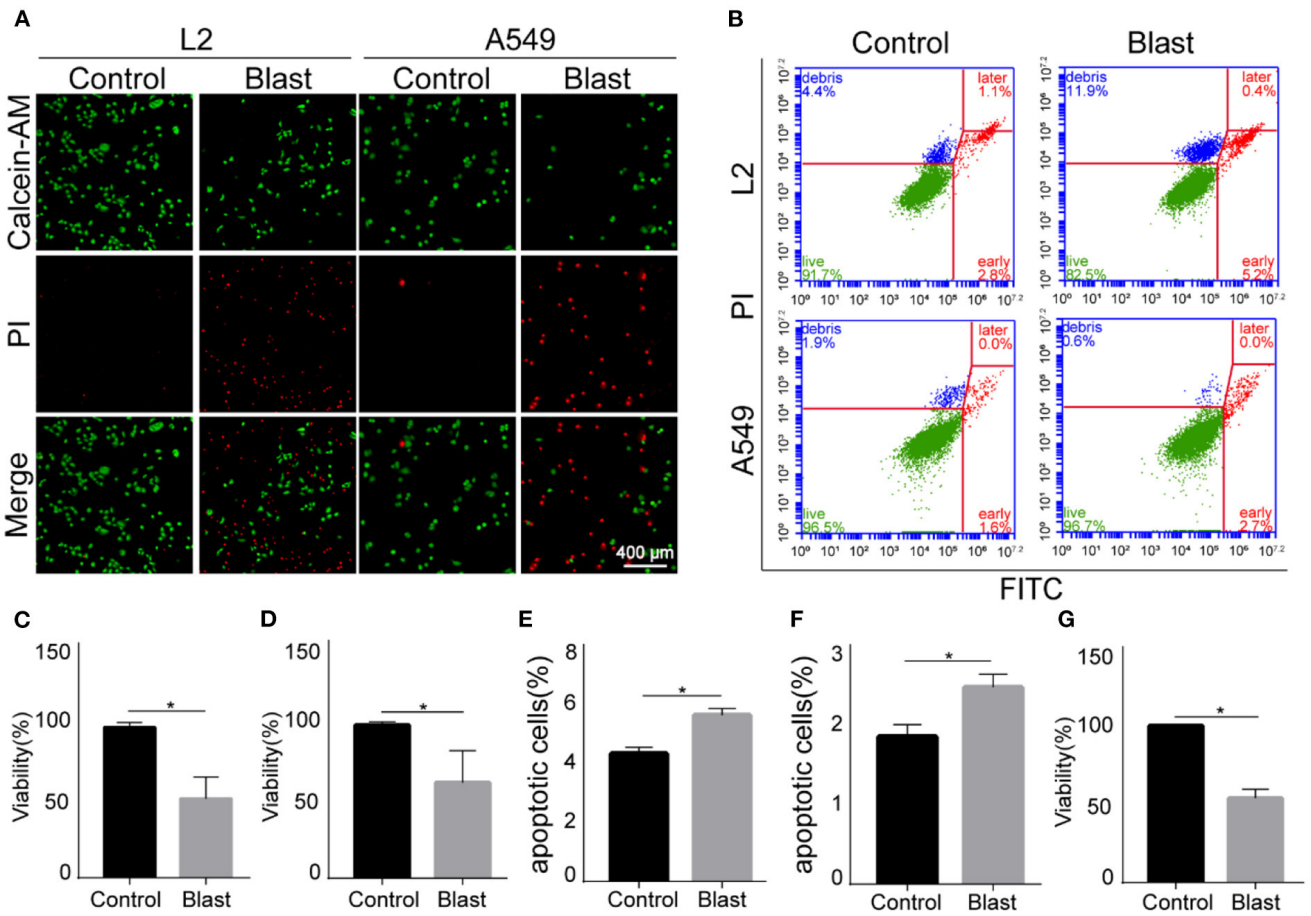
Effects of blast wave stimulation on viability and apoptosis of L2 and A549 cells. **(A)** Images of Calcein-AM/PI staining showing the living and membrane-ruptured L2 and A549 cells. Green, living cells; red, membrane-ruptured cells. **(B)** Dot plot showing the apoptosis of L2 and A549 cells. **(C, D)** Quantitative analysis and comparison of cell viability between the control and blast wave groups of L2 and A549 cells. **(E)** Comparison of L2 cell apoptosis between groups. **(F)** Comparison of A549 cell apoptosis between groups. **(G)** Comparison of A549 cell viability between groups. Data represent mean ± SD; *n* = 10. *P < 0.05. FITC, fluorescein isothiocyanate; PI, propidium iodide.

Based on above findings, the modeling conditions were set as 8-bar blast wave, 1,000-impulse at 10 Hz in 1 mL complete medium, 1 × 10^5^/mL modeling concentration, 6-h post stimulation culture time, and 1 × 10^4^ cell plating number. On the completion of above specified blast wave stimulation, we repeated the experiments using A549 cells. Compared with the control group, the viability of blast wave stimulated A549 cells decreased by 53.76 ± 1.92% (Figure 4G). Moreover, we explored the effect of specific modeling conditions on L2 cells and A549 cells. As shown in Figures 4A, C, D, the proportion of PI-stained cells increased at ~50% after blast wave stimulation, indicating the damage of cell membrane and decrease of cell viability. Besides, the apoptosis rate of L2 and A549 cells increased after exposing to blast wave (Figures 4B, E, F). These data indicated that the modeling condition could effectively introduce a median lethal dose to alveolar epithelial cells.

**Figure 5:**
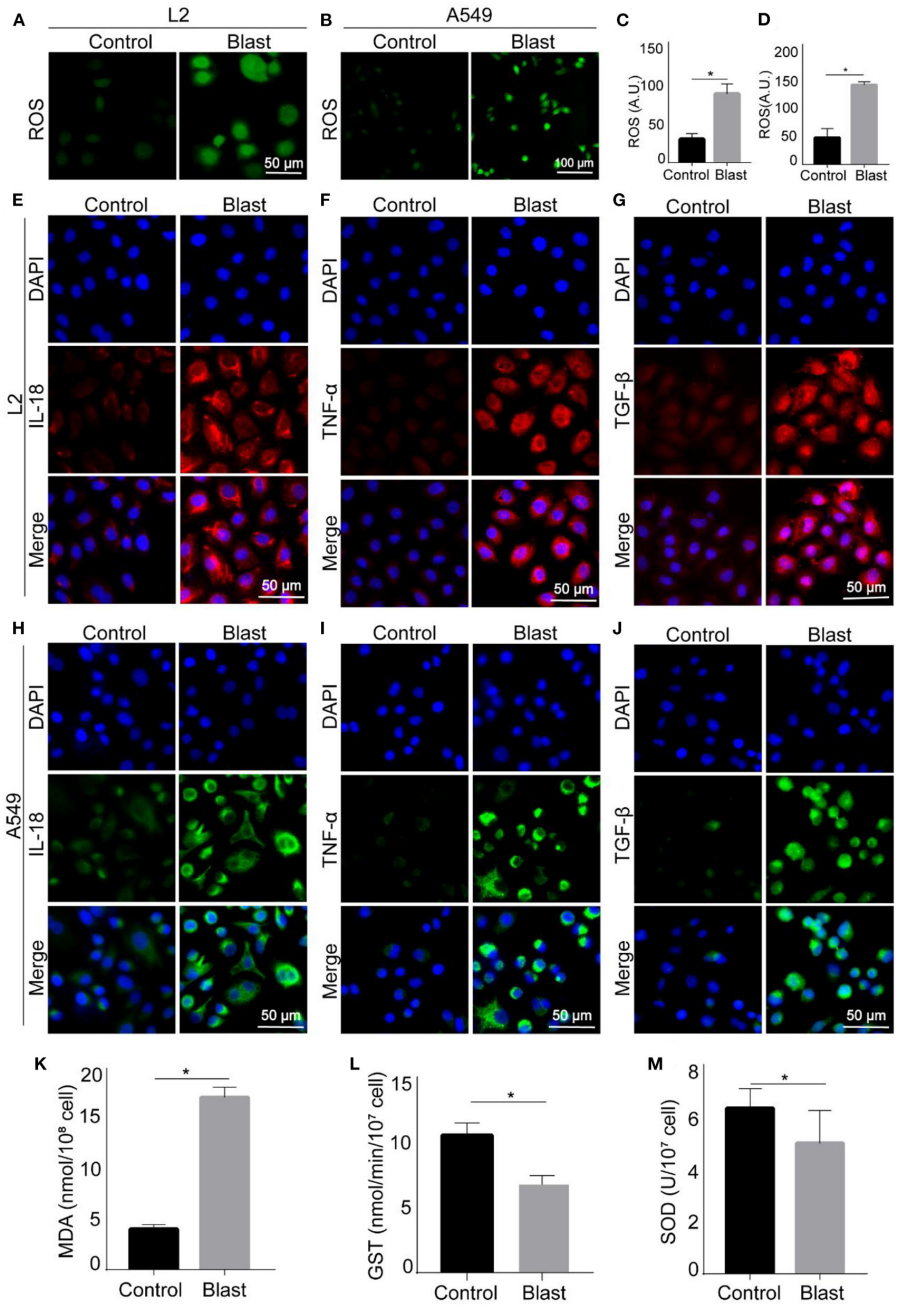
Oxidative stress and inflammatory response of blast wave stimulated alveolar epithelial cells. **(A, B)** Representative images of immunofluorescence staining showing the content of ROS in L2 and A549 cells. **(C, D)** Quantitative analysis and comparison of intracellular ROS between groups. **(E–G)** Representative images of immunofluorescence staining showing the expression of IL-18, TNF-α, and TGF-β in L2 cells. **(H–J)** Representative images of immunofluorescence staining showing the expression of IL-18, TNF-α, and TGF-β in A549 cells. **(K–M)** Quantitative analysis and comparison the intracellular MDA, GST, and SOD of L2 cells. Data represent mean ± SD; *n* = 10. *P < 0.05. IL-18, interleukin 18; TNF-α, tumor necrosis factor alpha; TGF-β, transforming growth factor beta; ROS, reactive oxide species; MDA, malonaldehyde; GST, Glutathione -S- transferase; SOD, superoxide dismutase.

Also, according to clinical manifestations of gas explosion-induced lung injury in the real world, we evaluated this *in vitro* blast model by examining the occurrence of oxidative stress and inflammation. As shown in Figures 5A–D, K–M, compared with the control group, the content of ROS increased in L2 and A549 cells after blast wave stimulation, whilst the anti-oxidative GST and SOD decreased in L2 cells. Figures 5E–J showed increased expression of inflammatory cytokines like IL-18, TNF-α, and TGF-β after blast wave stimulation. These data further confirmed that the modeling condition could induce similar alterations with that of gas explosion-induced human pulmonary injuries.

## 4. Discussion

Blast waves from gas explosion strike structures or impact in the immediate proximity of coal mine workers or structures housing them (21). The air blast shock wave in enclosed coal mining environment is the primary damage mechanism in an explosion which seriously threatens the life and health of coal mine workers (22). Due to the sudden and uncontrollable features of gas explosion, the mechanism of blast lung injury is still uncertain. Over the past years, several studies have tried to use the real roadway to establish *in vivo* blast lung injury model of rats (16, 23). Also, the shock tube is used to build the *in vitro* model of blast lung injury (24, 25). However, these two patterns of model establishing methods have high cost and relatively low successful rate in achieving a flexible and stable blast lung injury model because their experiment results are easy to be affected by different experimental conditions (26). In this study, a blast lung injury model based on alveolar epithelial cells is established using blast wave physiotherapy instrument, which can provide a stable, reliable and simple *in vitro* blast lung injury model, and this model is helpful for the mechanism exploration and clinical treatment research of related damages. When gas explodes, the blast wave of certain energy could cause mechanical damage to human alveolar epithelial cells. Moreover, the blast wave would be reflected when encountering obstacles, and the repeated blast wave would further aggravate the damage. In order to simulate the damage of human alveolar epithelial cells in real situations, we use different energy and impulse times to damage the cells, so as to find the most appropriate parameters. Finally, we find the parameters in detail for the model establishment: a blast wave physiotherapy type HM08CJ at 8 bar, 10 Hz blast wave on (1–5) × 10^5^ alveolar epithelial cells for 1,000 times.

In the process of blast lung injury model establishment, different parameter settings can produce varied modeling effects. To achieve the optimal modeling conditions, these parameters are explored one by one. For energy of blast wave which directly affected the severity of cell damage, we set different energy gradients and find that the cell viability increased slightly when the energy is 0.5 bar. Hence, we speculate that the slight blast wave might improve the cell viability to a certain extent and the blast wave intensity that the cells can tolerate is 0.5 bar, while increased energy of blast wave at 8 bar causes reduction of cell viability to (53.52 ± 2.86)%, which meet the preset modeling standard. Further, to explore the appropriate frequency of impulses, we set five gradients of the blast wave frequencies, and find that there is an obvious drop of cell viability when the impulse reach to 500 times, but the specific mechanism still needs to be discussed. At 10 Hz of impulse frequency, different times of impulse need different time, so we choose the 1,000 impulses since it takes the least time.

Since the cells undergo a repair process over time, the time of maintenance after modeling is also important. In order to observe the damage and repair of cells after stimulated by blast wave, six time points are set within 24 h, we find that the cell damage is most serious at 6 h, which is consistent with the results of Zhang (25) who utilizes shock tube to build cell damage model. After incubation for 12 h, the cell viability recovers to (74.33 ± 9.44)%. Similarly, some researchers report that the definition of primary blast lung injury is acute lung injury occurring within 12 h after blast wave exposure (27). Therefore, this model could simulate the process of blast lung injury with specific features of first injury then recovery. For reasons of this phenomenon, the cells could secrete various regulatory factors to repair itself during damage conditions, which for example could promote the degradation and re-utilization of damaged organelles through autophagy, thus maintaining the structure and function of cells (28). Besides, the cell viability declines again at 24 h, which may be due to the gradual reduction of nutrients in the culture plate over time, leading to the decline of cell viability.

Previous studies have shown that blast wave can induce free radical reaction to trigger oxidative stress, causing oxidative damage (29, 30). To compare the established model with gas explosion-induced lung injury in the real world, we examine the occurrence of oxidative stress by detecting intracellular ROS, MDA, GST, and SOD after the successful establishment of the blast lung injury model. We find that the content of ROS and MDA increase, meanwhile, the content of SOD and GST decrease, indicating that this model does effectively cause cell oxidative damage.

In addition to oxidative damage, blast waves are reported to trigger inflammatory response and apoptosis (31). Thereby, we examine the apoptosis and inflammatory responses of A549 and L2 cells. Among all inflammatory cytokines we concentrate, IL-18 plays an important role in early inflammatory response, it is reported to promote neutrophils aggregation and lympho T cell proliferation, and mediate cytotoxic reactions (32). TNF-α is located in the upstream of the inflammatory response and mediate the synthesis of various inflammatory cytokines (14). TGF-β can inhibit cell proliferation and induce cell differentiation or apoptosis (33, 34). Local inflammatory responses play an important role in tissue repair and regeneration, but excessive or persistent inflammatory responses might lead to systemic and uncontrolled inflammatory responses and distal organ failure (35). Apoptosis is a process in which cells automatically end their lives under certain physiological or pathological conditions under the control of intrinsic genetic mechanisms (36). Similarly, some researchers report that blast exposure leads to inflammation, oxidative stress, and apoptosis in mouse lungs (31).

This study provides a blast lung injury model which can definitely contribute to revealing the pathogenesis and clinical treatment of blast lung injury, the model is fast, simple, safe, and low-cost. However, there are not only blast injuries in explosion accidents, but also burns and inhalation injuries, but this model cannot simulate burns and inhalation injuries, which should further be improved in future studies.

## 5. Conclusion

In conclusion, an *in vitro* blast lung injury model is set up using a blast wave physiotherapy at 8 bar, 10 Hz blast wave by acting on (1–5) × 10^5^ alveolar epithelial cells for 1,000 times. The model is simple, safe, and stable, and can be used for studies of injury caused by gas explosion and blast-associated other external forces.

## Data availability statement

The raw data supporting the conclusions of this article will be made available by the authors, without undue reservation.

## Author contributions

CD: conceptualization, investigation, and writing—original draft. SH: data curation. MZ: formal analysis. YS and JZ: visualization. NL: software. LM: investigation. LT: method. WR: writing—review and editing. SY: supervision and writing—review and editing. LZ: conceptualization and writing—review and editing. All authors contributed to the article and approved the submitted version.

## References

[1] Le Roux R. The effect of the coal industry on indoor radon concentrations in eMalahleni, Mpumalanga Province of South Africa. Health Phys. (2022) 122:488–94. 10.1097/HP.0000000000001526 35085121

[2] Sun Z Li Y Wang M Wang X Pan Y Dong F. How does vertical integration promote innovation corporate social responsibility (ICSR) in the coal industry? A multiple-step multiple mediator model. PLoS ONE. (2019) 14:e0217250. 10.1371/journal.pone.0217250 31188841 PMC6561533

[3] Wei Q Hu B Fang H Zheng C Hou X Gao D. Effective approach with extra desorption time to estimate the gas content of deep-buried coalbed methane reservoirs: a case study from the Panji Deep Area in Huainan Coalfield, China. ACS Omega. (2022) 7:11240–51. 10.1021/acsomega.2c00142 35415329 PMC8992273

[4] Tong R Yang Y Ma X Zhang Y Li S Yang H. Risk assessment of miners' unsafe behaviors: a case study of gas explosion accidents in coal mine, China. Int J Environ Res Public Health. (2019) 16:1765. 10.3390/ijerph16101765 31109043 PMC6572149

[5] Deng J Qu J Wang QH Xiao Y Cheng YC Shu CM. Experimental data revealing explosion characteristics of methane, air, and coal mixtures. RSC Adv. (2019) 9:24627–37. 10.1039/C9RA04416G 35527867 PMC9069593

[6] Dong X Yao S Wu W Cao J Sun L Li H. Gas explosion-induced acute blast lung injury assessment and biomarker identification by a LC-MS-based serum metabolomics analysis. Hum Exp Toxicol. (2021) 40:608–21. 10.1177/0960327120960761 32969285

[7] Song Y Zhang Q. The quantitative studies on gas explosion suppression by an inert rock dust deposit. J Hazardous Mater. (2018) 353:62–9. 10.1016/j.jhazmat.2018.03.052 29635175

[8] Cernak I. The importance of systemic response in the pathobiology of blast-induced neurotrauma. Front Neurol. (2010) 1:151. 10.3389/fneur.2010.00151 21206523 PMC3009449

[9] Courtney A Courtney M. The complexity of biomechanics causing primary blast-induced traumatic brain injury: a review of potential mechanisms. Front Neurol. (2015) 6:221. 10.3389/fneur.2015.00221 26539158 PMC4609847

[10] Smith JE Garner J. Pathophysiology of primary blast injury. J Royal Army Med Corps. (2019) 165:57–62. 10.1136/jramc-2018-001058 30317218

[11] Izumi Y Nakashima T Masuda T Shioya S Fukuhara K Yamaguchi K. Suplatast tosilate reduces radiation-induced lung injury in mice through suppression of oxidative stress. Free Rad Biol Med. (2019) 136:52–9. 10.1016/j.freeradbiomed.2019.03.024 30930296

[12] Terasaki Y Suzuki T Tonaki K Terasaki M Kuwahara N Ohsiro J. Molecular hydrogen attenuates gefitinib-induced exacerbation of naphthalene-evoked acute lung injury through a reduction in oxidative stress and inflammation. Lab Investig J Tech Methods Pathol. (2019) 99:793–806. 10.1038/s41374-019-0187-z 30710119

[13] Liu YE Tong CC Zhang YB Cong PF Shi XY Liu Y. Chitosan oligosaccharide ameliorates acute lung injury induced by blast injury through the DDAH1/ADMA pathway. PLoS ONE. (2018) 13:e0192135. 10.1371/journal.pone.0192135 29415054 PMC5802901

[14] Liu Y Tong C Cong P Liu Y Shi X Shi L. Proteomic analysis revealed the characteristics of key proteins involved in the regulation of inflammatory response, leukocyte transendothelial migration, phagocytosis, and immune process during early lung blast injury. Oxidat Med Cell Long. (2021) 2021:8899274. 10.1155/2021/8899274 34007409 PMC8099533

[15] Smith JE. Blast lung injury. J R Naval Med Ser. (2011) 97:99–105. 10.1136/jrnms-97-99 22372014

[16] Dong X Wu W Yao S Cao J He L Ren H. Evaluation of gas explosion injury based on analysis of rat serum profile by ultra-performance liquid chromatography/mass spectrometry-based metabonomics techniques. BioMed Res Int. (2020) 2020:8645869. 10.1155/2020/8645869 32775446 PMC7407032

[17] Carrick FR McLellan K Brock JB Randall C Oggero E. Evaluation of the effectiveness of a novel brain and vestibular rehabilitation treatment modality in ptsd patients who have suffered combat-related traumatic brain injuries. Front Public Health. (2015) 3:15. 10.3389/fpubh.2015.00015 25699246 PMC4316606

[18] Lu CR Chen L Chen WB Dou CQ Liu R Huang ZQ. Absorbable bandage wrapping in treatment of severe blast liver injury: a miniature swine model. Chin Med J. (2011) 124:3757–61. 10.3760/cma.j.issn.0366-6999.2011.22.028 22340237

[19] Maitz A Haussner F Braumüller S Hoffmann A Lupu L Wachter U. Temporal-spatial organ response after blast-induced experimental blunt abdominal trauma. FASEB J Off Publ Feder Am Soc Exp Biol. (2021) 35:e22038. 10.1096/fj.202100995R 34748229

[20] Hayakawa K Esposito E Wang X Terasaki Y Liu Y Xing C. Transfer of mitochondria from astrocytes to neurons after stroke. Nature. (2016) 535:551–5. 10.1038/nature18928 27466127 PMC4968589

[21] Li J Qin Y Wang Z Xin Y. How to analyse the injury based on 24Model: a case study of coal mine gas explosion injury. Injury Prev J Int Soc Child Adoles Injury Prev. (2021) 27:542–53. 10.1136/injuryprev-2021-044281 34518337

[22] Tang C Zhang S Si Z Huang Z Zhang K Jin Z. High methane natural gas/air explosion characteristics in confined vessel. J Hazard Mater. (2014) 278:520–8. 10.1016/j.jhazmat.2014.06.047 25010457

[23] Tian LQ Guo ZH Meng WZ Li L Zhang Y Yin XH Lai F. The abnormalities of coagulation and fibrinolysis in acute lung injury caused by gas explosion Kaohsiung. J Med Sci. (2020) 36:929–36. 10.1002/kjm2.12262 32643870 PMC11896557

[24] Bass CR Meyerhoff KP Damon AM Bellizzi AM Salzar RS Rafaels KA. Drosophila melanogaster larvae as a model for blast lung injury. J Trauma. (2010) 69:179–84. 10.1097/TA.0b013e3181c42649 20173659

[25] Zhang Z Liang Z Li H Li C Yang Z Li Y. Perfluorocarbon reduces cell damage from blast injury by inhibiting signal paths of NF-κB, MAPK and Bcl-2/Bax signaling pathway in A549 cells. PLoS ONE. (2017) 12:e0173884. 10.1371/journal.pone.0173884 28323898 PMC5360309

[26] Gao K Shi L Li S Wen L. Propagation laws of discontinuous gas supply in the excavation roadway. PLoS ONE. (2022) 17:e0268453. 10.1371/journal.pone.0268453 35617247 PMC9135244

[27] McDonald Johnston A Ballard M. Primary blast lung injury. Am J Resp Crit Care Med. (2015) 191:1462–3. 10.1164/rccm.201501-0063IM 26075425

[28] Guillot L Nathan N Tabary O Thouvenin G Le Rouzic P Corvol H. Alveolar epithelial cells: master regulators of lung homeostasis. Int J Biochem Cell Biol. (2013) 45:2568–73. 10.1016/j.biocel.2013.08.009 23988571

[29] Tong C Liu Y Zhang Y Cong P Shi X Liu Y. Shock waves increase pulmonary vascular leakage, inflammation, oxidative stress, and apoptosis in a mouse model. Exp Biol Med. (2018) 243:934–44. 10.1177/1535370218784539 29984607 PMC6108052

[30] Scott TE Kirkman E Haque M Gibb IE Mahoney P Hardman JG. Primary blast lung injury: a review. Br J Anaesth. (2017) 118:311–6. 10.1093/bja/aew385 28203741

[31] Liu Y Tong C Xu Y Cong P Liu Y Shi L. CD28 deficiency ameliorates blast exposure-induced lung inflammation, oxidative stress, apoptosis, and T cell accumulation in the lungs *via* the PI3K/Akt/FoxO1 signaling pathway. Oxid Med Cell Long. (2019) 2019:4848560. 10.1155/2019/4848560 31565151 PMC6745179

[32] Yasuda K Nakanishi K Tsutsui H. Interleukin-18 in health and disease. Int J Mol Sci. (2019) 20:649. 10.3390/ijms20030649 30717382 PMC6387150

[33] Larson C Oronsky B Carter CA Oronsky A Knox SJ Sher D. TGF-beta: a master immune regulator. Exp Opin Therap Targets. (2020) 24:427–38. 10.1080/14728222.2020.1744568 32228232

[34] Bai X Yi M Jiao Y Chu Q Wu K. Blocking TGF-β signaling to enhance the efficacy of immune checkpoint inhibitor. OncoTargets Therapy. (2019) 12:9527–38. 10.2147/OTT.S224013 31807028 PMC6857659

[35] Vourc'h M Roquilly A Asehnoune K. Trauma-induced damage-associated molecular patterns-mediated remote organ injury and immunosuppression in the acutely ill patient. Front Immunol. (2018) 9:1330. 10.3389/fimmu.2018.01330 29963048 PMC6013556

[36] Elmore S. Apoptosis: a review of programmed cell death. Toxicol Pathol. (2007) 35:495–516. 10.1080/01926230701320337 17562483 PMC2117903

